# ^1^H NMR Metabolic Profiling and Meat Quality in Three Beef Cattle Breeds from Northeastern Thailand

**DOI:** 10.3390/foods11233821

**Published:** 2022-11-26

**Authors:** Chirasak Phoemchalard, Suthipong Uriyapongson, Tanom Tathong, Pitukpol Pornanek

**Affiliations:** 1 Department of Agriculture, Mahidol University, Amnatcharoen Campus, Amnatcharoen 37000, Thailand; 2 Department of Animal Science, Faculty of Agriculture, Khon Kaen University, Khon Kaen 40002, Thailand; 3 Department of Food Technology, Faculty of Agriculture and Technology, Nakhon Phanom University, Nakhon Phanom 48000, Thailand; 4 Department of Animal Science, Faculty of Natural Resources, Rajamangala University of Technology Isan, Sakon Nakhon Campus, Sakon Nakhon 47160, Thailand

**Keywords:** metabolic profiling, meat quality, beef

## Abstract

The increasing need for effective analytical tools to evaluate beef quality has prompted the development of new procedures to improve the animal sector’s performance. In this study, three beef breeds—Thai native (TN), crossbred Brahman × Thai native (BT), and crossbred Charolais × Brahman (CB)—were compared in terms of their physicochemical and metabolic profiles. The findings demonstrated that TN beef was lighter and tougher than other beef. Beef odor was stronger in BT. In addition, CB beef was the most tender and had the highest intramuscular fat content. Twenty-one different metabolites were found overall through NMR and chemometric approaches. The primary factors contributing to the difference in OPLS-DA loading plots were acetylcholine, valine, adenine, leucine, phosphocreatine, β-hydroxypyruvate, ethanol, adenosine diphosphate, creatine, acetylcholine, and lactate. The multivariate analysis indicated that these metabolites in beef cattle breeds could be distinguished using NMR spectroscopy. The results of this study provide valuable information on the quality and meat metabolites of different breeds. This could help in the development of a more accurate assessment of the quality of beef in future research.

## 1. Introduction

The increasing investigation of metabolic profiles using nuclear magnetic resonance spectroscopy (NMR) has resulted in a variety of animal, meat, milk, and food applications. The rapid development of NMR has dramatically contributed to understanding the quality-related components [1,2]. This technology has allowed scientists to examine the molecular composition of various raw materials in a non-invasive manner [3]. The high-throughput molecular analysis techniques can be used to identify a wide variety of metabolites and changes in the biological samples. It can be used to obtain accurate and comprehensive metabolic profiles of food products without the need for complex preparation. In several studies, NMR has been performed to analyze the effects of post-mortem aging [4,5,6,7,8,9], degrees of tenderness [10], pHu [11], irradiated beef [12], geographic origins [13], and beef authentication [14,15,16] on beef metabolic profiles.

There are four types of beef cattle in Thailand, namely Thai native cattle (TN), crossbred cattle, purebred cattle, and fattening beef cattle [17]. These cattle make up approximately 54.95%, 39.85%, 1.63%, and 3.57% of the total beef cattle population, respectively [17]. The Department of Livestock Development (Thailand) has recognized the following four breeds as Thai native cattle (*Bos indicus*): Kho-Khaolumpoon is from the northern region of the country, while the other three are from the central (Kho-Lan), northeastern (Kho-Isaan), and southern (Kho-Chon) regions [18]. For beef production in the northeast, traditional Kho-Isaan (TN) is supplied to open markets with unspecified meat quality. On the other hand, crossbred Brahman × Thai native (BT, *Bos indicus*) cattle are often grown for medium-quality markets by fattening culled cattle for four to six months to enhance body mass and fat [19,20]. Commercially produced crossbred Charolais and Brahman (CB, *Bos taurus × Bos indicus*) cattle target high-end markets, with a 1% market share [19,20].

There is currently no published data in the literature on using NMR-based metabolomics to detect TN, BT, and CB beef. Thus, this study aimed to investigate beef metabolomic profiles using a ^1^H NMR-based metabolomics analysis and beef quality characteristics and to consider the potential markers of TN, BT, and CB beef.

## 2. Materials and Methods

Samples of locally sourced and commercially produced beef products were obtained from various retail locations and local delivery outlets. The study did not involve the use of non-standard breeding techniques or animal testing. Therefore, it was also unnecessary to obtain approval from an ethical commission.

### 2.1. Meat Sample Preparation

Thirty authentic, fresh loin cuts from different types of beef were obtained during December 2021 at a local butcher’s shop approved by the Department of Livestock Development (Thailand) in Amnatcharoen, Sakon Nakhon, Nakhon Phanom, and Mukdahan Provinces, Thailand. These cuts were from TN (*n* =10), BT (*n* = 10), and CB (*n* = 10) beef cattle. Meat samples (approximately 500 g) were taken from different animals using the same anatomical cut. Loin cuts (*longissimus dorsi)*, from the 10th and 12th rib were cut, trimmed, boxed at a regular 4–5 °C temperature, and brought to the laboratory. The samples were refrigerated for 24 h before meat quality analysis. To prepare the NMR analysis, the remaining beef (100 g) was kept in the freezer (−80 °C).

### 2.2. Analysis of Meat Quality

#### 2.2.1. pH Measurement

After 24 h chilling, a pH electrode was used to quantify the pH levels of the meat in triplicate using a pH meter (HI99163, Hanna Instruments, Inc., Woonsocket, RI, USA) supplied with a FC2323 pH/temperature probe and a stainless steel blade. Before the measurement, the pH meter was adjusted to 4.0 (HI5004) and 7.0 (HI5007) standards [21].

#### 2.2.2. Instrumental Color Measurement

According to AMSA guidelines [21], the color of meat samples was measured in quintuplicate with a CR-400 colorimeter (Konica Minolta, Tokyo, Japan) using CIE L*a*b* values to measure brightness, redness, and yellowness. The readers were calibrated with the manufacturer’s whiteboard before each session. The flesh color of each sample was evaluated at five predetermined locations on the cutting surface.

#### 2.2.3. Water Holding Capacity (WHC) Measurement

For drip loss, meat samples were first weighed (W_i_), placed in plastic bags, hung, and refrigerated at 4 °C for 24 h. It was then removed, washed, and weighed again (W_f_) to calculate the loss using the following equation: drip loss (%) = [(W_i_ − W_f_)/W_i_] × 100. A 2.54 cm thick sample of each meat was weighed (W_i_), vacuumed, and sealed for cooking loss analysis. The sample’s core temperature reached 75 °C after boiling in a water bath at 80 °C. The samples were then removed, cooled, and stored overnight in a refrigerator at 4 °C. The sample is then removed, washed, and weighed (W_f_). Cooking loss values were calculated by considering the percentage of the initial weight of the sample, as follows: cooking loss (%) = [(W_i_ − W_f_)/W_i_] × 100. All WHC analyses were carried out in triplicate.

#### 2.2.4. Textural Properties of Beef

Further analyses of the Warner–Bratzler shear force (WBSF) and work of shear (WS) were then performed on the cooked beef samples. The WBSF and WS analysis were carried out using a methodology derived from the ASMA recommendations [22]. Each sample was cored using a tissue borer to collect six 1.27 cm cores in a direction perpendicular to the fibers. The USDA Warner–Bratzler Blade kit was attached to a TA.XTplus texture analyzer (Stable Micro Systems Ltd., Surrey, UK) to perform shear operations on the cores perpendicular to their myofiber orientation. The load cell and crosshead speed were 50 kg and 4 mm/s. A maximum force cut through the core (WBSF, kg/cm^2^) and the area under the curve (WS, kg·s) during a test were measured.

Another cooked beef sample was used for texture profile analysis (TPA) using the texture analyzer equipped with a 50 mm cylindrical probe. Various textural parameters were measured to evaluate its hardness, resilience, springiness, gumminess, adhesiveness, and cohesiveness. Next, the meat samples (1 × 1 × 1 cm^3^) were crushed twice to 3/4 of their initial thickness using a 50 kg transducer and crosshead rate of 1 mm/s [23]. The analysis results were automatically calculated using Exponent ver. 6.1.21.0. The triplicate readings were averaged after the samples were compressed.

#### 2.2.5. Macronutrients of Beef

The macronutrients, including the moisture, protein, fat, and ash of meat, were analyzed in triplicate by the AOAC procedures [24].

#### 2.2.6. Electronics Nose (E-Nose)

Meat flavors were analyzed using an E-NOSE instrument (Electronic Nose Co., Ltd., Bangkok, Thailand) equipped with eight types of MOS sensors (TGS 816, TGS 2600, TGS 823, TGS 2603, TGS 826, TGS 2610, TGS 2620, and TGS 2444). Sample preparation and E-NOSE conditions were conducted according to a previous research [23]. The sensors were tested at a temperature of 25 °C. The CIM NOSE 2.0 software (Electronic Nose Co., Ltd., Bangkok, Thailand) was used to calculate the sensor response percentages.

### 2.3. Metabolite Extraction, ^1^H NMR Analysis, Data Pre-Processing, and Metabolite Identification

About 100 g of frozen beef was thawed and minced, and 500 mg was collected and transported to Khon Kaen University International Phenome Laboratory (KKUIPL), the site where metabolic profiling of meat tissues was performed. At first, the tissue was extracted with chloroform and methanol (1:1). To separate the polar and lipophilic phases, the samples were centrifuged at 10,000× *g* for 15 min. Afterward, a rapid vacuum (Labconco, Kansas City, MO, USA) was used to extract them. Following tissue (100 mg) resuspension in HPLC-grade water (600 µL), they were vortexed until they dissolved completely. A fresh tube was used to transfer 540 µL of supernatant, and 60 µL of NMR buffer mixes made with 100% D_2_O containing 0.1 mM TSP, 1.5 M KH_2_PO_4_ buffer, and 2 mM NaN_3_ (Cambridge Isotype Laboratories, Tewksbury, MA, USA) were added. After spinning, 560 µL of excess was poured to a 5 mm NMR tube. Four quality control (QC) samples consisting of 70 µL of each extracted sample were taken, pooled, and processed as the study samples.

Data were acquired using 400 MHz nuclear magnetic resonance (NMR) (Bruker, Billerica, MA, USA). A typical 1-D pulse sequence was used to examine each sample at 300 K. Each ^1^H-NMR spectrum was achieved in 15 min. It was detected using a total of 64 scans, data points of 72 k, 20 ppm spectral width, 10 µs of a 90° pulse, and 4 s relaxation delay. The data collected during the study was analyzed using TopSpin 4.0, which is able to improve the performance of its peak alignment and normalization using the PQN method [25]. Various chemical shift referencing (δ^1^H 0.00 TSP peak) and baseline correction procedures were also performed. To minimize the possessions of the defective water suppression technique, the spectral data were cleaned of the water peak [25]. To confirm the effectiveness of the proposed method, the researchers used the computational technique known as statistical total correlation spectroscopy (STOCSY) [26] in MATLAB 2022b (MathWorks Inc., Natick, MA) to analyze the various resonances of interest. They then searched the databases of the Human Metabolome Database (HMDB), Kyoto Encyclopedia of Genes and Genomes (KEGG), Bovine Metabolome Database (BMDB), and Biological Magnetic Resonance Data Bank (BMRB).

### 2.4. Statistical Analysis

According to the information gathered from various animal breeds, a multivariate and univariate data analysis was accomplished. The data was assessed in a completely randomized design. All data collection were analyzed using the GLM procedure of SAS (SAS Institute, Inc., Cary, NC, USA) [27] through following models: *Y_ij_ = µ + Breed_i_ + ε_ij_*, where *Y_ij_* represents quality variables and metabolite concentration, *µ* represents the general mean, *Breed_i_* represents the TN, BT, and CB beef, and ε_ij_ represents the unsystematic variation. The Tukey HSD and Duncan’s new multiple range tests (DMRT) were accomplished on different breeds after analysis of variance (ANOVA) to compare their statistical changes. The difference (*p* < 0.05) between the means of all traits was regarded as significant. The relative concentrations of metabolites were stored in MetaboAnalyst 5.0 [28]. An unsupervised (principal component analysis, PCA) method with Pareto-scaling was then performed to analyze the components and discriminants followed by a supervised method (orthogonal signal correction–projection to latent structures–discriminant analysis, OPLS-DA). “The Pareto scaling is the square root of the standard deviation; this scaling strategy minimizes the relative importance of high values while preserving a portion of the data structure maintaining a more precise value than autoscaling” [29]. Before running the OPLS-DA, two data groups were chosen, while another group and QC group were omitted. The predictability and fitness of the OPLS-DA models were strongminded by the R^2^X, R^2^Y, and Q^2^Y. In addition, a permutation test (100 times), in which a *p*-value less than 0.05 results, further confirmed the models’ accuracy. The gap between R^2^Y and Q^2^Y must not reach 0.3, or Q^2^Y intercepts below 0.05 could also be viewed as an indicator of model validity avoiding overfitting [30]. A variable importance in projection (VIP > 1, *p* < 0.05, and FDR < 0.05) charts from the models were applied to identify the most distinctive compounds. Beef metabolites as recognized and confirmed in the public database were submitted, normalized, and analyzed using pathway analysis tool in MetaboAnalyst 5.0, using *Bos taurus* KEGG pathways based on a comprehensive knowledge base (http://www.metaboanalyst.ca, accessed on 1 August 2022) [28]. MetaboAnalyst 5.0 was also used to conduct a Pearson’s correlation. All data were presented as LS mean and standard deviation.

## 3. Results and Discussion

### 3.1. Beef Quality

Meat quality is a vital factor that consumers rely on to ensure they receive the best possible product [31]. The physical characteristics of beef loins from different breeds (CB, TN, and BT) are presented in Table 1. Effects of breed on the quality characteristics, including pH, a*, b*, chroma, hue, drip loss, cooking loss, adhesiveness, springiness, and cohesiveness of meat were not significant (*p* > 0.05). Meat from TN had more lightness than BT and CB beef (*p* < 0.05). Changes in L*a*b* values may have been altered by pH [32,33]. The strongest correlation to lean maturity is L* value, while a* and b* values had a stronger correlation with muscle pH [32]. Meat color levels, on the other hand, were comparable to beef produced in Thailand [34,35]. Compared to BT and TN beef, CB beef was the most tender (*p* < 0.05). Meat from TN beef was harder (*p* < 0.05) due to higher degrees of shear force, hardness, gumminess, and chewiness compared with other beef. The WBSF had a positively associated with the L* (r = 0.42, *p* < 0.05), moisture (r = 0.65, *p* < 0.001), and protein (r = 0.49, *p* < 0.01), but a negative relationship with fat (r = −0.64, *p* < 0.001). The tougher meat in TN and BT may be partly explained by breed, age, and production systems resulting in higher calpastatin activity [19,36]. Because the average shear force was less than 4.2 kg/cm^2^, it was intermediately tender (3.9 < WBSF < 4.6 kg/cm^2^) [37]. However, the present WBSF was lower than those reports of [19,35,38] in TN, [19,34] in BT, and [19] in CB beef. The difference in the results might be due to the different test conditions. For instance, the shear force machine and crosshead speed used might have an impact. This is supported by Wheeler at al., who indicated that difference in protocol had a greater effect on varying shear values [39]. Moreover, tenderness can be caused by the postmortem factors [40] and the expression of various molecular factors, including heat shock proteins, structural proteins, metabolic proteins, and oxidative stress proteins [41,42]. Meat composition was influenced (*p* < 0.05) by animal breeds. The moisture and ash level of TN beef was high, but the fat content was low. Additionally, beef from BT had more protein and less ash, while beef from CB had more fat but less protein and ash. In comparison, almost all of the chemical compositions of beef were similar to other studies [19,34,35,43,44] except for fat. The meat from the CB group had the most intramuscular fat (IMF), since it was from a European breed and was finished with high-energy diets for a longer time. Both the TN and BT groups were allowed to graze on natural forages, but the BT group was sometimes fed a concentrated diet. These results agreed with [19], who found that grain-finished cattle had a greater IMF than forage-finished beef. Additionally, the factors that affect meat quality include the animal’s genetics, nutrition, environment, and production status. These factors can also affect the animal’s fat, lean, and connective tissue components [31].

In terms of raw meat odor characteristics, the average sensing response values of beef samples on eight metal oxide semiconductor (MOS) sensors are shown in Table 2. Beef displayed a very high sensitivity to the organic gases (Sensor 3), trimethylamine and methyl mercaptan (Sensor 4), ethanol, isobutane, and ammonia (Sensor 5), and organic solvent and alcohol vapors (Sensor 7). However, there were no observable differences (*p* > 0.05) in the responses of sensors 4 and 8 (ammonia). The lowest response rates (*p* < 0.05) of sensors 1 (butane, methane, propane), 2 (smoke, alcohol), 3, 5, 6 (propane, isobutane, methane, respectively), and 7 were found in TN Beef. Several studies have shown that an E-NOSE with MOS sensors can differentiate between different types of meat by detecting their odor [45,46,47,48,49]. The increased Sensor 3 response was in line with [23], which indicated that meat contains more volatile organic compounds (VOCs). In this study, BT beef’s VOCs differed from those of TN and CB beef. The responses of sensors 2, 5, and 7 were more remarkable in BT beef, indicating that BT beef had a more pungent odor overall.

### 3.2. H-NMR Metabolic Profiling of Beef

**Figure 1:**
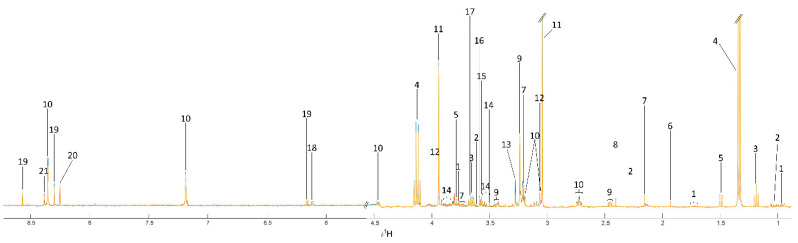
^1^H NMR CPMG median spectra of each group, metabolite names listed in Table 3.

A representative ^1^H-NMR analysis of the various compounds found in beef extracts revealed that there were 21 primary metabolites (Figure 1 and Table 3) as follows: leucine, valine, ethanol, lactate, alanine, acetate, acetylcholine, succinate, carnitine, unknown 1, creatine, phosphocreatine, trimethylamine-n-oxide, unknown 2, 3-hydroxyisobutyrate, glycine, β-hydroxy pyruvate, unknown 3, adenosine diphosphate, adenine, and unknown 4. Comparing metabolites between selected breeds, Tukey post hoc pairwise testing was used. The concentrations of several metabolites in the beef samples from various breeds varied considerably. Only unknown 4 metabolites were different between TN and BT beef. The TN and CB beef had different levels of leucine, valine, ethanol, lactate, acetylcholine, creatine, phosphocreatine, β-hydroxy pyruvate, adenosine diphosphate, adenine, and unknown 4. In addition, most of the measurable metabolite levels, including leucine, valine, ethanol, lactate, creatine, phosphocreatine, trimethylamine-n-oxide, unknown 2, β-hydroxy pyruvate, unknown 3, adenosine diphosphate, and adenine, revealed substantial variations between the BT and CB beef. The amounts of the various metabolites found in the beef extracts of three cattle breeds compared using DMRT after ANOVA are detailed in Table 4. Eleven of the identified metabolites namely leucine, valine, ethanol, lactate, acetylcholine, creatine, phosphocreatine, trimethylamine-n-oxide, β-hydroxy pyruvate, adenosine diphosphate, and adenine, and three unidentified metabolites including unknown 2, unknown 3, and unknown 4, were significantly different among TN, BT, and CB beef. Of these, lactate and creatine were the most abundant.

The levels of these compounds were strongly influenced by various factors including production technique, feeding regimen, and environment. However, some of these effects cannot be fully explained [13]. The high levels of creatine and lactate are consistent with earlier studies in beef [4,5,13]. Creatine is essential to the energy metabolism of muscular tissue [50,51]. It can improve meat quality by reducing the accumulation of lactic acid in the body [52]. Levels of the acid in meat can negatively affect meat quality and contribute to water-holding capacity [53], but do not in the current study.

### 3.3. Multivariate Statistical Analysis and Pathways Analysis of Metabolites

**Figure 2:**
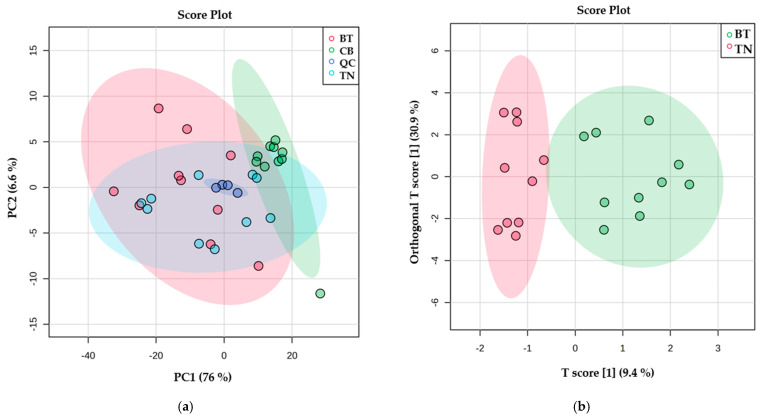

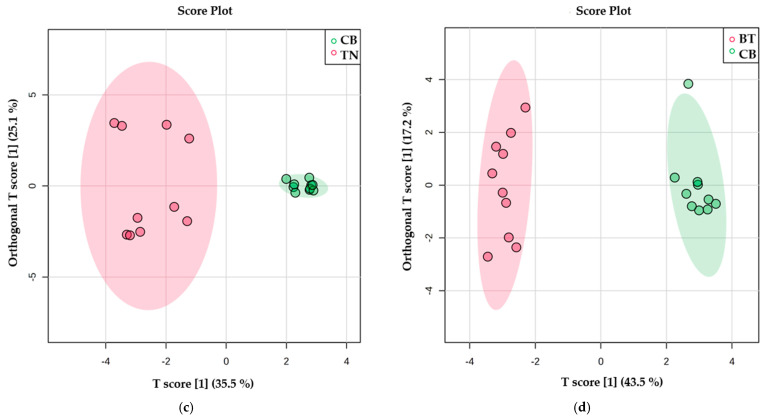
PCA (**a**) score plot of PC1 and PC2, and pairs of OPLS-DA (**b**–**d**) score plots of orthogonal T score [1] versus T score [1] produced from beef metabolites. (**a**) Overall, R^2^X = 0.563, Q^2^X = 0.288; (**b**) TN vs BT, R^2^X = 0.705, R^2^Y = 0.839, Q^2^Y = 0.152; (**c**) TN vs CB, R^2^X = 0.606, R^2^Y = 0.938, Q^2^Y = 0.866; (**d**) BT vs CB, R^2^X = 0.693, R^2^Y = 0.987, Q^2^Y = 0.941. Abbreviations are as follows: Thai native beef (TN), Brahman × Thai native beef (BT), Charolais × Brahman beef (CB), and Quality Control (QC).

An unsupervised method known as PCA is used to characterize multiple data sets. It can carry out its function without knowing about the data set beforehand [54]. The OPLS-DA method uses multivariate data to discriminate groups. It is an improvement over the PLS-DA approach. The PCA and OPLS-DA methods were used to generate comparative interpretations and visual representations of the metabolic variations between beef breeds by R^2^X, Q^2^X, R^2^Y, and Q^2^Y values. The PCA and OPLS-DA score plots used to analyze the relative concentrations of beef extracts are displayed in Figure 2a. The principal components of the PC1 and PC2 explained 76.6% and 6.6% of the variation. However, the other groups were not entirely separated by unsupervised PCA (R^2^X = 0.563, Q^2^X = 0.288). In Figure 2b, the OPLS-DA model showed good performance in discriminating between TN and BT beef. Three orthogonal factors and one predictive factor were used to formulate the model. The key parameters of this model, R^2^X = 0.705, R^2^Y = 0.839 (*p* = 0.03), and Q^2^Y = 0.152 (*p* = 0.07), were good predictors of model fit. However, its predictive capabilities were not strong. The disparity between R^2^Y and Q^2^Y was excessive (0.687), and their intercepts were 0.673 and −0.884 (not shown), respectively, indicating that great predictability results from excessive data fitting. Using *p*-value (*p* < 0.05), VIP (>1), and FDR (<0.05), only the single most identifying variable (unknown 4) was prioritized for comparing Thai native beef with crossbred Brahman beef (Table 5). Figure 2c reveals the OPLS-DA score plot of TN and CB beef. The model was constructed using a combination of one of each predictive and orthogonal component.

The variation value in the OPLS-DA model was explained by the R^2^X = 0.606, R^2^Y = 0.938 (*p* < 0.01) and Q^2^Y = 0.866 (*p* < 0.01) values, which indicated a good fit and prediction. Furthermore, there was a difference of 0.072 between R^2^Y and Q^2^Y, and their intercepts were 0.30 and −0.366 (not shown), respectively. The original model overfitted if Q^2^Y intercept was more than 0.05 [55]. Our analyses, therefore, indicate statistical validity. The 10 potential metabolites (Table 5) that could distinguish between TN and CB beef were prioritized by the VIP and *p*-value groups. Compared to crossbred Charolais, Thai native beef had higher concentrations of β-hydroxypyruvate, ethanol, adenosine diphosphate, creatine, acetylcholine, and lactate, while the concentrations of valine, leucine, phosphocreatine, and adenine were the opposite. The OPLS-DA score plot in Figure 2c showed significant differences between BT and CB beef. The model was constructed using two orthogonal components and one predictive component. The variation value was explained using the R^2^X = 0.693, R^2^Y = 0.987 (*p* < 0.01) and Q^2^Y = 0.941 (*p* < 0.01) values. The values of R^2^Y and Q^2^Y differed by 0.046 and had intercepts of 0.442 and −0.685 (not shown), respectively, proposing the model is reliable. These results showed excellent discrimination between crossbred Brahman and crossbred Charolais beef, along with very strong fit and prediction. The 10 potential metabolites (Table 5) were analyzed as described previously. Leucine, adenine, valine, phosphocreatine, and unknown 2 were, respectively, higher in CB than in BT beef. On the other hand, adenosine diphosphate, ethanol, creatine, β-hydroxypyruvate, and lactate were the opposite. These results agree with several metabolomic studies which utilized a multivariate approach to analyze large sets of data, allowing them to discriminate between redundant and relevant information [13,56,57]. In addition, the non-targeted technique can be used to visualize the differences in the metabolism patterns of different beefs [13], milks [56], cheeses [57,58,59], ducks [60], and other food products from different origins.

Major metabolic pathways (*p* < 0.05) uncovered by *Bos taurus* library-based analysis are presented in Table S1 and Figure S3. Because unknown 4 metabolites did not match any KEGG database, pathway analysis comparing TN and BT beef did not reveal a statistically significant difference. The main pathways in TN and CB beef (Figure S3b) were (1) arginine and proline metabolism, (2) valine, leucine, and isoleucine biosynthesis, (3) aminoacyl-tRNA biosynthesis, (4) valine, leucine, and isoleucine degradation, (5) purine metabolism, (6) pantothenate and CoA biosynthesis, (7) glycerophospholipid metabolism, and (8) glycine, serine, and threonine metabolism. In comparison to TN vs. CB (Figure S3c), glycolysis/gluconeogenesis and pyruvate metabolism pathways were two more significant metabolic pathways for BT and CB. Therefore, these metabolic pathways were affected by animal breed, suggesting that the metabolisms of various cattle breeds may differ. The most important genetic groups that are known to differentiate themselves from one another are amino acids, such as biosynthesis leucine, isoleucine, and valine, metabolism of alanine, aspartate and glutamate, and metabolism of glutamine and glutamate [10].

### 3.4. Relationship of Metabolites and Meat Quality

Relationships between metabolites and meat quality parameters in beef are shown in Figure S1. The ultimate pH was negatively associated with the unknown 2 (r = −0.40, *p* < 0.05), while the surface color and cooking loss did not correspond with any of the metabolites. The weak positive correlation between the amount of drip loss and the unknown 1 (r = 0.38, *p* < 0.05), unknown 2 (r = 0.37, *p* < 0.05), and a weak negative correlation with alanine (r = −0.39, *p* < 0.05) variables were observed. In this study, unknown 4 was positively correlated with meat tenderness in terms of shear force (r = 0.46, *p* < 0.05), work of shear (r = 0.49, *p* < 0.01), and hardness (r = 0.49, *p* < 0.01), demonstrating that the modification of unknown 4 metabolites has moderately altered the tenderness of meat. In addition, using pattern hunter analysis, as seen in Figure S2, a moderate correlation between shear force and unknown 4 (r = 0.46, *p* = 0.011), glycine (r = −0.35, *p* = 0.055), acetylcholine (r = −0.33, *p* = 0.07), and β-hydroxypyruvate (r = −0.30, *p* = 0.106) can be observed. The lowering of these negatively associated metabolites will enhance the rate of toughness. Meat contains various metabolites that can be associated with odor. Some of these, including lactate (r = −0.48, *p* = 0.01), adenosine diphosphate (r = −0.49, *p* = 0.01), ethanol (r = −0.45), creatine (r = −0.43), and β-hydroxypyruvate (r = −0.40), were moderately negatively correlated to sensor 1, while a weak or moderate correlation with valine (r = 0.38), phosphocreatine (r = 0.39,) and adenine (r = 0.44) were positive. Thus, the response of the MOS-1 sensor rose when lactate and β-hydroxypyruvate decreased, whereas valine, phospho-creatine, and adenine increased. Moreover, alanine (MOS 2–7), carnitine (MOS 2, 5), unknown 2 (MOS 5), unknown 4 (MOS 2–3, 5–7), phosphocreatine (MOS 8), and β-hydroxypyruvate (MOS 8) were statistically correlated.

Meat pH is generally associated with moisture and shear force. However, it was negatively associated with protein levels and water holding capacity [61]. Thus, if animals have lower ultimate pH values, they are more prone to drip loss and lighter color [62]. However, the final pH in this experiment was not associated with the beef quality or any other known parameters. Although lightness was not linked to any metabolic changes, it was positively associated with certain physical properties, such as cooking loss (r = 0.38, *p* < 0.05), shear force (r = 0.42, *p* < 0.01), hardness (r = 0.56, *p* < 0.01), and moisture (r = 0.41, *p* < 0.01), but was negatively associated with fat (r = −0.37, *p* < 0.05), sensor 2 (r = −0.46, *p* < 0.01), sensor 3 (r = −0.41, *p* < 0.05), sensor 5 (r = −0.46, *p* < 0.05), sensor 6 (r = −0.38, *p* < 0.05), sensor 7 (r = −0.46, *p* < 0.05), and sensor 8 (r = −0.43, *p* < 0.05). Meat quality is linked to the presence of lactic acid in the meat. This condition affects various aspects of the meat, such as its pH, color, and tenderness [63]. Nevertheless, there was no relationship between cutting strength and lactate levels (r = 0.02, *p* > 0.05) in this study. The weak relationship between most metabolites and shear force was similar to a previously reported work [10]. However, samples with less unknown 4 in this study were found to be more tender. These findings suggest that unknown 4 may be a possible indicator for beef tenderness. To properly comprehend the connection between the quantity of unknown 4 and beef tenderness, further study is necessary. A dozen of the compounds found in beef are commonly associated with the smell of meat. These mainly include amino acids and hydroxy acids. Interestingly, free amino acids are important in the quality of meat, as they contain the precursor substance for the flavor and taste of the meat [64]. Jeong et al. [65] noted that among the metabolites found in high-marbled meat were carnitine, glucose, lactate, and carnosine. The presence of these potential markers in beef can be linked to various taste-related pathways, such as amino acid metabolism and carbohydrate metabolism.

## 4. Conclusions

We concluded that metabolomics technology with chemometric technics could be helpful in identifying the origins of beef products in the meat industry. In addition, it can be used to monitor the quality of the product. This study used ^1^H NMR analysis, and is the first metabolomic investigation of Thai native, crossbred Brahman, and crossbred Charolais beef cattle raised in Thailand. Our findings revealed that only one metabolite distinguished Thai native and crossbred Brahman. In contrast, numerous metabolites are possible biomarkers for discriminating Thai native from crossbred Charolais and crossbred Brahman from crossbred Charolais. In conclusion, we suggest that using metabolomic and chemometric analysis could be useful in the evaluation of beef quality. In the future, we hope to use metabolomics to improve the quality of meat in producing high-quality products. 

## Figures and Tables

**Table 1:** Physicochemical characteristics of beef loins (M. Longissimus dorsi) from different breeds.

Items	TN	BT	CB	*p*-Value
pH	5.61 ± 0.09	5.56 ± 0.16	5.52 ± 0.05	0.253
Meat color				
L*	39.25 ± 3.27 ^a^	34.63 ± 3.36 ^b^	34.19 ± 3.13 ^b^	0.003
a*	15.16 ± 3.13	15.08 ± 1.89	14.87 ± 1.88	0.961
b*	8.27 ± 1.62	8.38 ± 0.60	8.78 ± 0.73	0.550
C*	17.33 ± 3.11	17.29 ± 1.63	17.27 ± 1.91	0.998
h*	28.98 ± 5.83	29.33 ± 3.82	30.70 ± 2.13	0.632
Water holding capacity				
Drip loss (%)	11.15 ± 2.12	9.89 ± 2.45	9.77 ± 1.31	0.256
Cooking loss (%)	26.78 ± 2.85	23.40 ± 4.77	23.05 ± 3.59	0.072
Shear values				
Shear force (kg/cm^2^)	4.33 ± 1.01 ^a^	3.97 ± 1.30 ^a^	2.30 ± 0.65 ^b^	<0.001
Work of shear (kg·s)	10.85 ± 1.87 ^a^	9.64 ± 3.49 ^a^	5.19 ± 1.28 ^b^	<0.001
Texture profile analysis				
Hardness (g)	1298.03 ± 611.26 ^a^	774.07 ± 260.01 ^b^	710.85 ± 203.36 ^b^	0.005
Adhesiveness (g·s)	−4.43 ± 5.70	−4.50 ± 4.02	−10.35 ± 13.60	0.245
Springiness	0.13 ± 0.07	0.09 ± 0.01	0.12 ± 0.04	0.156
Cohesiveness	0.76 ± 0.27	0.70 ± 0.06	0.75 ± 0.08	0.644
Gumminess	995.86 ± 606.16 ^a^	536.02 ± 176.79 ^b^	550.60 ± 154.66 ^b^	0.016
Chewiness	149.71 ± 162.91 ^a^	48.31 ± 21.93 ^b^	66.33 ± 31.09 ^a,b^	0.060
Resilience	0.29 ± 0.05 ^b^	0.30 ± 0.04 ^a^	0.36 ± 0.06 ^a^	0.005
Chemical composition				
Moisture (%)	74.46 ± 0.32 ^a^	73.58 ± 0.39 ^b^	64.87 ± 1.37 ^c^	<0.001
Protein (%)	21.99 ± 0.30 ^b^	23.17 ± 0.16 ^a^	20.57 ± 0.13 ^c^	<0.001
Fat (%)	2.12 ± 0.13 ^b^	2.43 ± 0.30 ^b^	13.40 ± 1.73 ^a^	<0.001
Ash (%)	1.43 ± 0.50 ^a^	0.82 ± 0.19 ^b^	1.17 ± 0.52 ^a,b^	0.014

^a,b,c^ denote the degree of significance (*p* < 0.05) represented by the variations between the following breeds: Thai native beef (TN), Brahman × Thai native beef (BT), and Charolaise × Brahman beef (CB).

**Table 2:** Sensing response (%) of beef loins (M. Longissimus dorsi) from different breeds.

Items	TN	BT	CB	*p*-Value
MOS-1 (TGS 816)	8.28 ± 1.47 ^a,b^	7.55 ± 1.61 ^b^	9.34 ± 2.15 ^a^	0.028
MOS-2 (TGS 2600)	8.97 ± 4.16 ^c^	17.78 ± 3.16 ^a^	12.68 ± 3.93 ^b^	<0.001
MOS-3 (TGS 823)	24.35 ± 12.28 ^b^	41.68 ± 7.00 ^a^	30.71 ± 8.85 ^b^	<0.001
MOS-4 (TGS 2603)	27.79 ± 13.01	32.85 ± 5.21	31.07 ± 5.66	0.399
MOS-5 (TGS 826)	17.88 ± 5.43 ^c^	31.83 ± 4.77 ^a^	23.28 ± 6.51 ^b^	<0.001
MOS-6 (TGS 2610)	7.06 ± 2.22 ^b^	9.70 ± 1.47 ^a^	8.63 ± 1.53 ^a^	0.004
MOS-7 (TGS 2620)	15.31 ± 5.66 ^c^	24.34 ± 3.65 ^a^	19.61 ± 4.05 ^b^	<0.001
MOS-8 (TGS 2444)	8.03 ± 1.25	9.54 ± 1.47	11.43 ± 1.29	0.131

^a,b,c^ denote the degree of significance (*p* < 0.05) represented by the variations between the following breeds: Thai native beef (TN), Brahman × Thai native beef (BT), and Charolaise × Brahman beef (CB). Different MOS sensor types are as follows: MOS-1 (butane, methane, and propane), MOS-2 (smoke, alcohol), MOS-3 (organic solvent vapors), MOS-4 (methyl mercaptan, trimethylamine), MOS-5 (isobutane, ethanol, and ammonia), MOS-6 (propane, isobutane, and methane), MOS-7 (alcohol, organic solvent), and MOS-8 (ammonia) [23].

**Table 3:** List of metabolites, chemical shift, and their pair wise test.

No.	Metabolite	Chemical Shift (Multiplicity)	*p*-Value		
			TN/BT	TN/CB	BT/CB
1	Leucine	0.9516 (t); 1.725 (m); 3.727 (t)	0.999	<0.001	<0.001
2	Valine	0.9917 (dd); 1.037 (d); 2.28 (m); 3.632 (d)	0.558	<0.001	<0.001
3	Ethanol	1.1707 (t); 3.65 (q)	0.835	<0.001	<0.001
4	Lactate	1.3281 (d); 4.103 (q)	0.456	0.012	<0.001
5	Alanine	1.485 (d); 3.772 (q)	0.734	0.986	0.637
6	Acetate	1.931 (s)	0.197	0.734	0.570
7	Acetylcholine	2.156 (s); 3.205 (s); 3.742 (t)	0.187	0.005	0.252
8	Succinate	2.4064 (s)	0.938	0.107	0.199
9	Carnitine	2.44 (dd); 3.239 (s); 3.424 (m)	0.054	0.738	0.225
10	Unknown 1	2.7038 (dt); 3.055 (dd); 3.195 (t); 4.454 (t); 7.188 (s); 8.35 (d)	0.881	0.627	0.349
11	Creatine	3.0432 (s); 3.941 (s)	0.763	<0.001	<0.001
12	Phosphocreatine	3.0638 (s); 3.974 (s)	1.000	<0.001	<0.001
13	Trimethylamine-N-oxide	3.2764 (s)	0.111	0.776	0.026
14	Unknown 2	3.503 (s); 3.527 (m); 3.727 (m); 3.846 (m); 3.974 (m)	0.106	0.364	0.005
15	3-Hydroxyisobutyrate	1.055 (d); 3.5597 (dd); 3.643 (dd)	0.746	0.104	0.361
16	Glycine	3.5782 (s)	0.930	0.960	0.801
17	β-hydroxypyruvate	3.668 (s)	0.998	<0.001	<0.001
18	Unknown 3	6.1105 (d)	0.080	0.335	0.003
19	Adenosine diphosphate	4.19 (m); 6.1549 (d); 8.30 (s); 8.567 (s)	0.802	<0.001	<0.001
20	Adenine	8.2509 (s)	0.954	<0.001	<0.001
21	Unknown 4	8.3845 (s)	0.008	0.049	0.713

Abbreviations are as follows: s, singlet; d, doublet; t, triplet; dd, doublet of doublets; dt, doublet of triplets; q, quartet; m, multiplet. Different beef breeds are as follows: Thai native beef (TN), Brahman × Thai native beef (BT), and Charolaise × Brahman beef (CB). The *p*-values were derived from the Tukey post hoc pairwise comparison tests [27].

**Table 4:** Relative concentrations (mAU) of beef metabolites using 400 MHz ^1^H-NMR spectra (0–10 ppm).

Metabolite	TN	BT	CB	*p*-Value
Leucine	1.76 ± 0.87 ^b^	1.75 ± 0.45 ^b^	3.58 ± 0.43 ^a^	<0.001
Valine	1.78 ± 0.84 ^b^	2.13 ± 0.50 ^b^	4.71 ± 0.84 ^a^	<0.001
Ethanol	21.18 ± 2.79 ^a^	21.77 ± 2.63 ^a^	16.29 ± 1.22 ^b^	<0.001
Lactate	521.18 ± 103.58 ^a^	575.09 ± 116.52 ^a^	384.22 ± 72.17 ^b^	0.001
Alanine	21.21 ± 3.65	19.94 ± 5.03	21.48 ± 2.00	0.627
Acetate	13.72 ± 10.04	8.85 ± 2.34	11.65 ± 2.63	0.223
Acetylcholine	26.33 ± 12.51 ^a^	20.06 ± 4.84 ^a,b^	14.41 ± 0.95 ^b^	0.008
Succinate	14.19 ± 9.50	13.18 ± 5.86	7.93 ± 2.78	0.096
Carnitine	8.96 ± 3.06	6.67 ± 1.51	8.26 ± 1.23	0.060
Unknown 1	6.79 ± 2.58	7.33 ± 3.44	5.75 ± 0.46	0.371
Creatine	394.25 ± 92.32 ^a^	416.88 ± 81.01 ^a^	257.69 ± 19.67 ^b^	<0.001
Phosphocreatine	11.52 ± 2.96 ^b^	11.48 ± 2.50 ^b^	23.24 ± 5.54 ^a^	<0.001
Trimethylamine-N-oxide	30.17 ± 15.01 ^b^	45.40 ± 22.90 ^a^	25.20 ± 6.98 ^b^	0.027
Unknown 2	3.79 ± 1.45 ^a^	2.55 ± 1.07 ^b^	4.61 ± 1.41 ^a^	0.006
3-Hydroxyisobutyrate	11.75 ± 1.79	10.54 ± 2.30	8.25 ± 0.83	0.117
Glycine	12.97 ± 5.91	12.07 ± 7.16	13.64 ± 2.17	0.816
β-hydroxypyruvate	17.84 ± 1.79 ^a^	17.89 ± 2.30 ^a^	14.18 ± 0.83 ^b^	<0.001
Unknown 3	2.68 ± 0.74 ^b^	3.51 ± 0.95 ^a^	2.16 ± 0.74 ^b^	0.004
Adenosine diphosphate	4.25 ± 1.95 ^a^	4.63 ± 1.27 ^a^	1.31 ± 0.32 ^b^	<0.001
Adenine	8.31 ± 4.40 ^b^	8.72 ± 2.92 ^b^	18.20 ± 1.57 ^a^	<0.001
Unknown 4	6.71 ± 0.77 ^b^	9.33 ± 2.17 ^a^	8.70 ± 2.07 ^a^	0.008

^a,b^ denote the degree of significance (*p* < 0.05) represented by the variations between the following breeds: Thai native beef (TN), Brahman × Thai native beef (BT), and Charolaise × Brahman beef (CB).

**Table 5:** A possible biomarker for separating meat metabolites from three distinct breeds.

Breeds	Metabolites	VIP	*p*-Value	Coefficient	FDR
TN vs. BT	Unknown 4	2.4946	0.002	−0.7669	0.043
TN vs. CB	Valine	1.5107	<0.001	−0.9001	<0.001
	Leucine	1.4722	<0.001	−0.8771	<0.001
	Phosphocreatine	1.4166	<0.001	−0.8440	<0.001
	Adenine	1.4126	<0.001	−0.8416	<0.001
	β-hydroxypyruvate	1.3993	<0.001	0.8337	<0.001
	Ethanol	1.3449	<0.001	0.8013	<0.001
	Adenosine diphosphate	1.2807	<0.001	0.7631	<0.001
	Creatine	1.2655	<0.001	0.7540	<0.001
	Acetylcholine	1.1157	0.003	0.6647	<0.001
	Lactate	1.0973	0.008	0.6538	0.016
BT vs. CB	Leucine	1.3805	<0.001	0.9107	<0.001
	Adenine	1.3545	<0.001	0.8936	<0.001
	Adenosine diphosphate	1.3480	<0.001	−0.8893	<0.001
	Valine	1.3396	<0.001	0.8837	<0.001
	Ethanol	1.2745	<0.001	−0.8407	<0.001
	Creatine	1.2687	<0.001	−0.8369	<0.001
	Phosphocreatine	1.2681	<0.001	0.8365	<0.001
	β-hydroxypyruvate	1.1678	<0.001	−0.7704	<0.001
	Lactate	1.1131	<0.001	−0.7343	<0.001
	Unknown 2	1.0333	0.002	0.6817	0.004

Abbreviations are as follows: VIP, variable importance in projection; FDR, false recovery rate; TN, Thai native beef; BT, Brahman × Thai native beef; CB, Charolaise × Brahman beef.

## Data Availability

This article presents all relevant information and procedures. Any additional requests should be contacted to the corresponding author.

## Supplementary Materials

The following supporting information can be downloaded at: https://www.mdpi.com/article/10.3390/foods11233821/s1, Figure S1: Correlation heatmaps between relative metabolite concentrations and beef quality, Figure S2: Top 21 shear force-associated metabolites using Pearson’s correlation in MetaboAnalyst 5.0 software, Figure S3: Metabolic pathways mapping of beef metabolites between beef breeds. Table S1: Major metabolic pathways (*p* < 0.05) uncovered by *Bos taurus* (KEGG) library-based analysis.
