# Supplementary material for: 1H NMR Metabolic Profiling and Meat Quality in Three Beef Cattle Breeds from Northeastern Thailand
Source: Foods. 2022 Nov 26;11(23):3821. doi: 10.3390/foods11233821 (PMC9736620; doi:10.3390/foods11233821)
Supplement: Supplementary file 1 [file foods-11-03821-s001.zip › foods-1996989-supplementary.pdf]

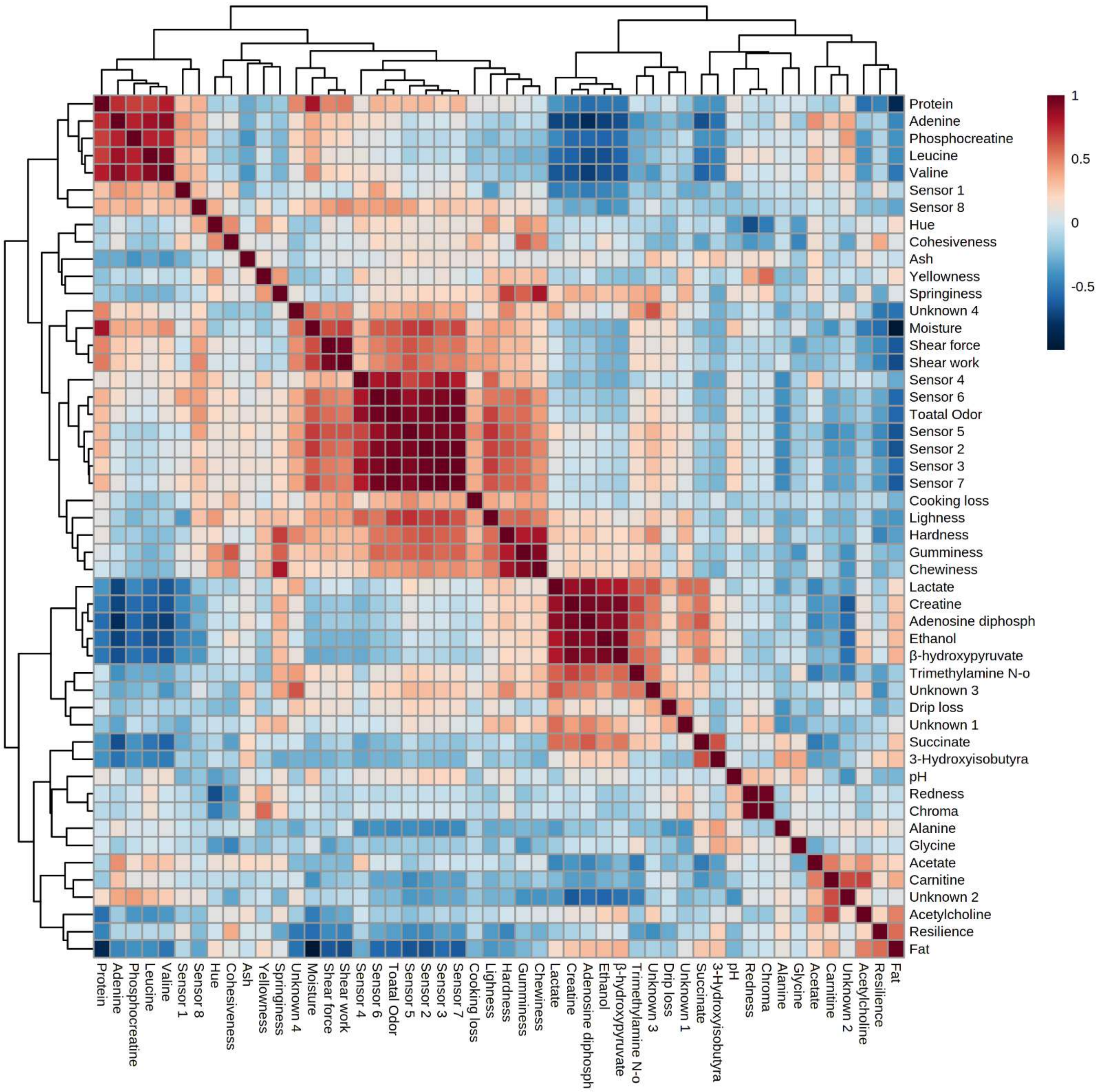

Figure S1. Correlation heatmaps between relative metabolite concentrations and beef quality: TN, Thai native beef; BT, Brahman × Thai native beef; CB, Charolaise × Brahman beef. Metabolites data were pareto scaled and analyzed with Pearson correlation in MetaboAnalyst 5.0 software. The top-right color key on the map indicates the expression values where the highly negative correlations are represented by dark blue, highly positive ones by dark red.

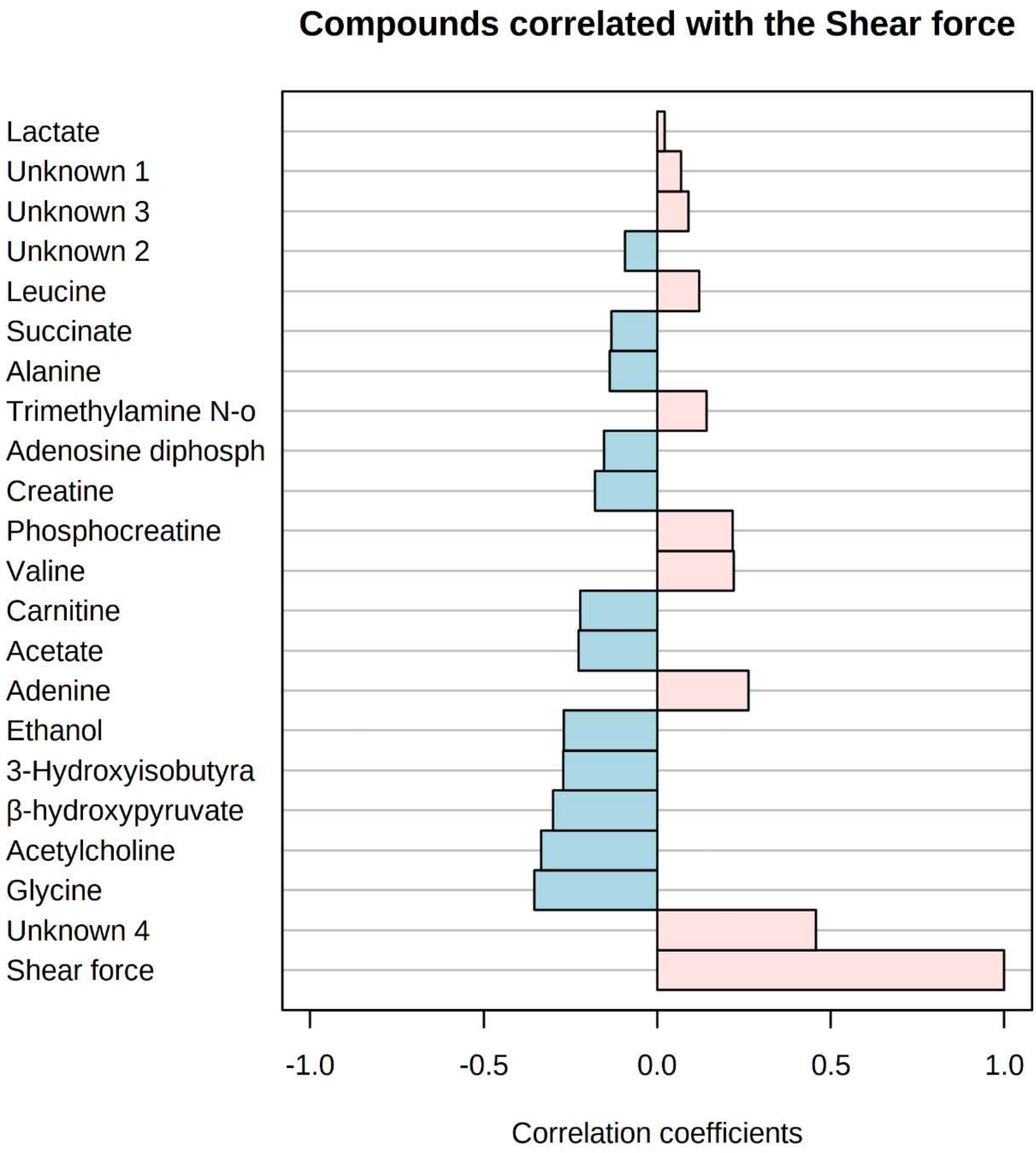

Figure S2. Top 21 shear force-associated metabolites using Pearson correlation in MetaboAnalyst 5.0 software.

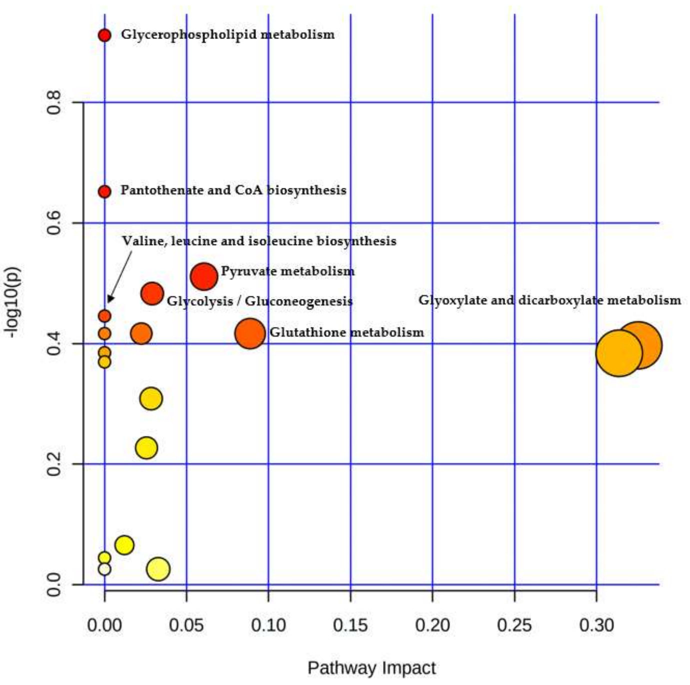

(a)

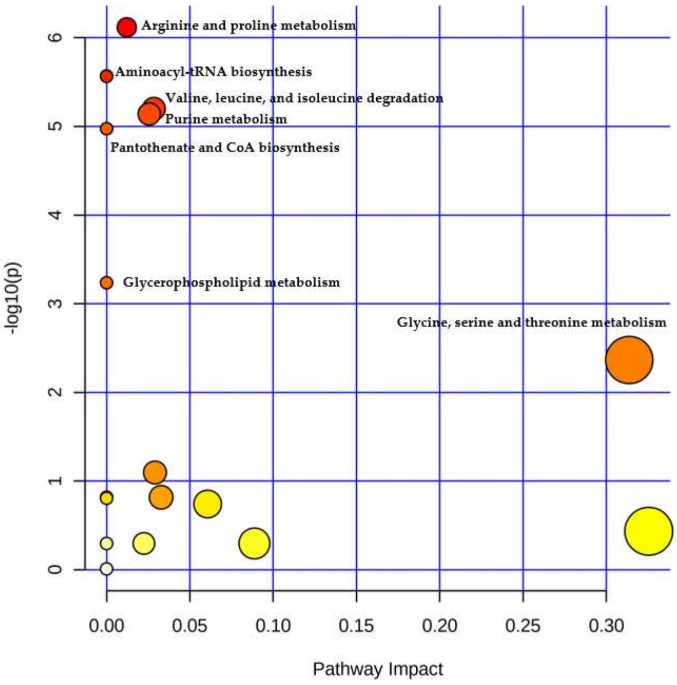

(b)

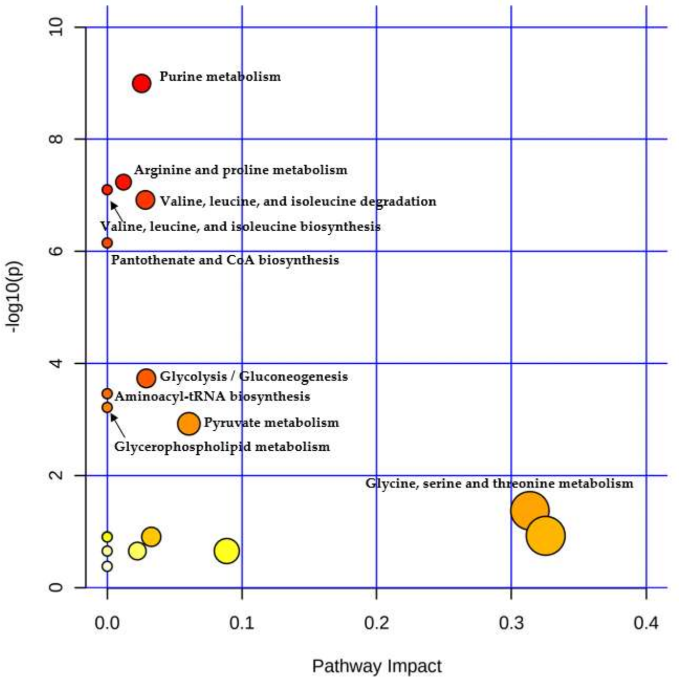

(c)

Figure S3. Metabolic pathways mapping of beef metabolites between TN and BT (**a**, $p > 0.05$), TN and CB (**b**, $p < 0.05$), and BT and CB (**c**, $p < 0.05$) revealed by *Bos taurus* (KEGG) library-based analysis using the MeatboAnalyst 5.0 software. The mapping displays the different pathways that were compared based on their *p* values in the topological and enrichment analyses. The larger and darker bubble plot shows the higher *p* values.

Table S1. Major metabolic pathways ($p < 0.05$) uncovered by *Bos taurus* (KEGG) library-based analysis.

Pathway Name	Match Status	p	-log(p)	Holm p	FDR	Impact
TN vs BT [a]						
-						
TN vs CB						
Arginine and proline metabolism	2/38	7.63×10^{-7}	6.12	1.45×10^{-5}	1.45×10^{-5}	0.01
Valine, leucine, and isoleucine biosynthesis	2/8	2.70×10^{-6}	5.56	4.86×10^{-5}	1.73×10^{-5}	0.00
Aminoacyl-tRNA biosynthesis	4/48	2.73×10^{-6}	5.56	4.86×10^{-5}	1.73×10^{-5}	0.00
Valine, leucine, and isoleucine degradation	3/40	6.34×10^{-6}	5.19	1.01×10^{-4}	2.75×10^{-5}	0.03
Purine metabolism	2/66	7.24×10^{-6}	5.14	1.09×10^{-4}	2.75×10^{-5}	0.03
Pantothenate and CoA biosynthesis	1/19	1.06×10^{-5}	4.97	1.49×10^{-4}	3.35×10^{-5}	0.00
Glycerophospholipid metabolism	1/36	5.80×10^{-4}	3.24	0.007	0.0015	0.00
Glycine, serine, and threonine metabolism	3/34	0.0043	2.36	0.052	0.0102	0.31
BT vs CB						
Purine metabolism	2/66	1.01×10^{-9}	8.99	1.92×10^{-8}	1.92×10^{-8}	0.03
Arginine and proline metabolism	2/38	5.83×10^{-8}	7.23	1.05×10^{-6}	5.05×10^{-7}	0.01
Valine, leucine, and isoleucine biosynthesis	2/8	7.97×10^{-8}	7.09	1.36×10^{-6}	5.05×10^{-7}	0.00
Valine, leucine, and isoleucine degradation	3/40	1.21×10^{-7}	6.92	1.94×10^{-6}	5.74×10^{-7}	0.03
Pantothenate and CoA biosynthesis	1/19	7.07×10^{-7}	6.15	1.06×10^{-5}	2.69×10^{-6}	0.00
Glycolysis / Gluconeogenesis	3/26	1.85×10^{-4}	3.73	0.0025	5.85×10^{-4}	0.03
Aminoacyl-tRNA biosynthesis	4/48	3.46×10^{-4}	3.46	0.0045	9.39×10^{-4}	0.00
Glycerophospholipid metabolism	1/36	6.09×10^{-4}	3.22	0.0073	0.0014	0.00
Pyruvate metabolism	2/22	0.0012	2.92	0.0131	0.0025	0.06
Glycine, serine, and threonine metabolism	3/34	0.0423	1.37	0.4239	0.0805	0.31

FDR, False recovery rate; TN, Thai native beef; BT, Brahman × Thai native beef; CB, Charolaise × Brahman beef

[a] Nineteen pathways were analyzed, and none of them showed statistical significance ($p > 0.05$).
